# Vitamin D deficiency and pregnancy outcomes: LC-MS/MS-based evaluation in Southwest China

**DOI:** 10.3389/fmed.2025.1711506

**Published:** 2026-01-22

**Authors:** Mao Zheng, Yali Wang, Ning Zou, Jianfei E., Yu Zou

**Affiliations:** 1 Department of Clinical Laboratory, Deyang People's Hospital, Deyang, China; 2 Department of Blood Transfusion, Deyang People's Hospital, Deyang, China

**Keywords:** 25(OH)D, adverse outcomes, LC-MS/MS, pregnancy, vitamin D deficiency

## Abstract

**Background:**

Vitamin D insufficiency is a global public health concern, particularly among Chinese pregnant women, yet southwest-China data using gold-standard LC-MS/MS are scarce.

**Methods:**

A retrospective cohort study was conducted on 2,742 pregnancies delivered in Deyang. Serum 25(OH)D2 and 25(OH)D3 quantified by LC-MS/MS (CV < 7%). Univariate and multivariate logistic regression analyses were used to identify risk factors for vitamin D deficiency and assess its associations with pregnancy outcomes. Efficacy of vitamin D supplementation was also compared.

**Results:**

The median serum 25(OH)D concentration was 16.3 ng/ml, with 62.5% of participants classified as vitamin D deficient (<20 ng/ml). Early pregnancy (≤12 weeks) carried 3.37-fold higher deficiency risk than late pregnancy (≥25 weeks). Early deficiency was associated with gestational hypothyroidism (OR = 1.32, *P* = 0.048). Paradoxically, late deficiency inversely correlated with preterm birth (OR = 0.32, *P* = 0.014). One-month supplementation: D3-800 IU raised 25(OH)D by +28.4 ng/ml, outperforming D2-800 IU (+14.6 ng/ml, *P* < 0.001).

**Conclusions:**

Vitamin D deficiency affects three-fifths of gravidae in Deyang. Early pregnancy represents the highest-risk window and predicts hypothyroidism. Late deficiency unexpectedly linked to lower preterm birth warrants causal clarification. D3 is the preferred supplement.

## Introduction

Vitamin D is an essential fat-soluble secosteroid that regulates intestinal calcium/phosphorus absorption, bone mineralization, and systemic homeostasis via the ubiquitously expressed vitamin D receptor (VDR), with pleiotropic effects on metabolism, immunity, and carcinogenesis (1). Pregnancy amplifies vitamin D demand to support maternal health and fetal development, making deficiency a critical concern-maternal insufficiency increases neonatal vitamin D inadequacy risk over eight-fold (2, 3). Despite global attention to maternal health, vitamin D deficiency remains highly prevalent among pregnant women worldwide (54% globally) and in China, where early pregnancy deficiency exceeds 70% nationally and 60% in urban centers like Shanghai (4–7). China's recent fertility policy reforms further underscore the urgency of optimizing maternal vitamin D status to improve pregnancy outcomes.

However, key research gaps persist. First, regional disparities in vitamin D status remain understudied, particularly in southwest China (e.g., Deyang). This region's unique geographical (low latitude), climatic (humid, frequent cloud cover), and lifestyle factors may influence cutaneous vitamin D synthesis differently than other Chinese regions, yet data specific to this population are lacking. Second, the association between maternal vitamin D deficiency and gestational complications/adverse outcomes remains controversial, partly due to limitations of prior studies relying on immunoassays-these methods are prone to cross-reactivity with vitamin D metabolites, leading to inaccurate status assessment (8). In contrast, liquid chromatography-tandem mass spectrometry (LC-MS/MS) offers superior specificity and sensitivity for quantifying circulating vitamin D metabolites (e.g., 25-hydroxyvitamin D2/3), enabling precise evaluation of true vitamin D status (9). Recent studies have also highlighted the broader implications of vitamin D deficiency, including its association with altered physiological parameters in diverse populations. For example, vitamin D deficiency correlates with abnormal platelet function in COVID-19 patients (10), suggesting potential immunomodulatory and inflammatory pathways that may also impact pregnancy.

To address these gaps, this study aimed to: (a) characterize vitamin D status and its determinants among pregnant women in Deyang, southwest China; and (b) investigate the association between accurately measured vitamin D deficiency and gestational complications/adverse outcomes. The findings will inform region-specific supplementation strategies, addressing a critical unmet need for evidence-based maternal health interventions in southwest China.

## Methods

### Study population and design

This was a single-center, population-based, observational retrospective study. The study cohort included pregnant women who underwent routine antenatal examinations at Deyang People's Hospital (Deyang, China; 31°N, 104°E) from January 2023 to December 2023. Approximately 5,000 pregnant women receive regular prenatal care at this hospital annually. Comprehensive data were extracted from the hospital's electronic medical record system and laboratory information management system.

Inclusion criteria: (a) maternal age 20–45 years; (b) local residency ≥ 2 years (to ensure exposure to regional environmental/lifestyle factors); (c) serum 25(OH)D measured via LC-MS/MS during gestation; (d) successful parturition at the study hospital. Exclusion criteria: (a) incomplete medical records; (b) pre-existing conditions affecting vitamin D metabolism (e.g., parathyroid disorders, chronic renal insufficiency, hepatic diseases); (c) maternal/paternal tobacco use or alcohol abuse before/during pregnancy; (d) fetal congenital anomalies or intrauterine fetal demise.

### Data collection

Demographic, anthropometric, obstetric, and clinical data were collected by qualified healthcare practitioners during early pregnancy antenatal registration. Key variables included: (a) demographics: age, ethnicity, occupation, residential location; (b) anthropometrics: pre-pregnancy weight/height (to calculate BMI); (c) obstetric history: last menstrual period, prior adverse pregnancy history (embryonic arrest, spontaneous abortion, stillbirth); (d) clinical data: pre-existing comorbidities, nutritional supplementation use (including vitamin D-containing multivitamins); (e) parturition and neonatal data: gestational complications, gestational age at delivery, mode of delivery, adverse maternal/neonatal outcomes, neonatal anthropometrics (birth weight/length), Apgar scores.

### Stratifications

Pre-pregnancy BMI: underweight (< 18.5 kg/m^2^), normal weight (18.5–23.9 kg/m^2^), overweight (≥24.0 kg/m^2^) (11); Season of 25(OH)D sampling: spring (Feb–Apr), summer (May–Jul), autumn (Aug–Oct), winter (Nov–Jan); Maternal age: 20–24 years, 25–29 years, 30–34 years, ≥35 years; Gestational age: early pregnancy ( ≤ 12 weeks), middle pregnancy (13–24 weeks), late pregnancy (≥25 weeks); Economic status: high (professional/managerial occupations), middle (administrative/service industry workers or small-scale freelancers); low (manual laborers, agricultural workers, or unemployed individuals).

For the supplementation subgroup: pregnant women diagnosed with vitamin D deficiency in early pregnancy were prescribed vitamin D2 (Dalian Aquatic Pharmaceutical Co., Ltd., 400 IU/capsule) or vitamin D3 (Beijing Langdi Pharmaceutical Co., Ltd., 200 IU/capsule) per medical recommendations. Serum 25(OH)D levels were longitudinally monitored via LC-MS/MS during follow-up.

### 25 (OH)D quantification via LC-MS/MS

Fasting venous blood samples were collected in separation gel vacuum tubes, centrifuged to isolate serum, and analyzed using an AB SCIEX Triple Quad™ 4500 MD LC-MS/MS system (USA). 25(OH)D2/D3 concentrations were quantified using isotope-labeled internal standards and a linear calibration curve (25(OH)D2: 0.1–200 ng/ml; 25(OH)D3: 0.15–400 ng/ml). Total 25(OH)D was the sum of 25(OH)D2/D3. Intra-assay and inter-assay CV were < 5.0% and < 7.0%, respectively. The laboratory is accredited by the China National Accreditation Service for Conformity Assessment and participates in external quality assessment programs. Vitamin D status was classified as sufficient (≥30 ng/ml), insufficient (20–29 ng/ml), or deficient (< 20 ng/ml) (12, 13).

### Handling of confounding factors

Potential confounders were identified based on literature review and clinical relevance, including maternal age, pre-pregnancy BMI, season of sampling, occupation (proxy for socioeconomic status), residential location (urban/rural, proxy for sunlight exposure access), and multivitamin supplementation (proxy for dietary vitamin D intake). These variables were included in the multivariable logistic regression models to adjust for their potential influence on vitamin D status and pregnancy outcomes. Socioeconomic status and dietary intake were not directly measured due to limitations in retrospective data availability, but validated proxies (occupation, supplementation) were used to minimize residual confounding. Sunlight exposure was indirectly accounted for via seasonal stratification and residential location, as direct exposure data (e.g., outdoor activity duration) were not recorded in electronic medical records.

Missing data handling: missing data accounted for < 3% of the total dataset and were managed via multiple imputation with 5 imputed datasets. The imputation model was constructed using all variables included in the final multivariate regression analyses (maternal age, pre-pregnancy BMI, gestational age, pregnancy pattern, adverse pregnancy history, routine multivitamin supplementation, place of residence, and sampling season). The imputed datasets were derived from the original study cohort of 2,742 pregnant women, with no external population data incorporated. All imputed datasets were validated for consistency with the original dataset's baseline characteristics (e.g., median 25(OH)D concentration, prevalence of vitamin D deficiency) before being integrated into the final statistical analyses to maintain statistical power and minimize bias.

### Definition of adverse pregnancy outcomes

Adverse pregnancy outcomes were predefined and extracted from standardized hospital records (validated via clinical documentation review by two independent researchers, with discrepancies resolved via consensus):

**Gestational complications**: gestational hypothyroidism (thyroid-stimulating hormone > 4.0 mIU/L with normal free thyroxine), gestational diabetes mellitus (abnormal oral glucose tolerance test), gestational anemia (hemoglobin < 110 g/L), intrahepatic cholestasis of pregnancy (elevated serum bile acids + pruritus), gestational hypertension (systolic blood pressure ≥140 mmHg and/or diastolic blood pressure ≥90 mmHg after 20 weeks of gestation).

**Adverse delivery/neonatal outcomes**: prematurerupture of membrane (rupture of membranes before 37 weeks), preterm birth (delivery at < 37 weeks of gestation), fetal distress (abnormal fetal heart rate pattern + meconium-stained amniotic fluid), term low birth weight (birth weight < 2,500 g at ≥ 37 weeks of gestation), postpartum hemorrhage (blood loss ≥ 500 ml after vaginal delivery or ≥ 1,000 ml after cesarean section).

### Pre-specification of primary pregnancy outcomes

Based on prior evidence, clinical relevance, and the protocol approved by the ethics committe for this retrospective study (approval number: 2023-04-101-K01), one gestational complication and one parturition outcome were predefined during the study design phase:

- gestational complication: association between early pregnancy vitamin D deficiency and gestational hypothyroidism. This was selected due to robust biological plausibility—vitamin D receptors share structural homology with thyroid hormone receptors, and prior studies have linked vitamin D deficiency to impaired immune regulation and increased risk of autoimmune thyroid diseases (14, 15). Additionally, both conditions are highly prevalent in Chinese pregnant women (7, 16), making this association clinically actionable.
- parturition outcome: association between late pregnancy vitamin D deficiency and preterm birth. Preterm birth is a leading cause of neonatal morbidity and mortality globally, with an estimated 15 million cases annually. Despite conflicting results in existing literature (17–19), the potential role of vitamin D in regulating uterine contractility and inflammatory pathways (key drivers of preterm birth) warranted clarification using gold-standard LC-MS/MS measurements.

Other secondary gestational complications (gestational diabetes mellitus, gestational anemia, intrahepatic cholestasis of pregnancy, gestational hypertension) and secondary parturition outcomes (uterine atony, cesarean delivery, postpartum hemorrhage, postpartum anemia, oligohydramnios, prematurerupture of membrane, fetal distress, term low birth weight) were exploratory, designed to generate hypotheses rather than test pre-specified research questions.

### Statistical analysis

Statistical analyses were performed using IBM SPSS Statistics Version 26.0. Continuous variables were presented as median and interquartile range [M (Q1, Q3)] and compared via the Kruskal–Wallis test. Categorical variables were expressed as frequencies and percentages, with intergroup comparisons using Pearson's chi-square test. Multivariable logistic regression models were constructed to: (1) identify independent risk factors for vitamin D deficiency; (2) assess associations between early pregnancy vitamin D deficiency and gestational complications; (3) evaluate associations between late pregnancy vitamin D deficiency and adverse outcomes. All models included predefined confounders (maternal age, BMI, season, residential location, supplementation). Multicollinearity testing: variance inflation factors (VIF) were calculated for all variables in regression models; a VIF < 5 indicated no significant multicollinearity. To mitigate the risk of overfitting: (1) stepwise forward selection based on the Akaike Information Criterion (AIC) was used to select independent variables, with only variables that significantly improved model fit (ΔAIC > 2) retained; (2) model goodness-of-fit was evaluated using the Hosmer–Lemeshow test (*P* > 0.05 indicating good fit) and area under the receiver operating characteristic curve (AUC > 0.7 indicating acceptable discriminative ability). All statistical tests were two-tailed, with significance set at *P* < 0.05. To account for the risk of type I error from multiple comparisons (e.g., subgroup analyses of vitamin D status determinants), the Bonferroni correction was applied to adjust the significance threshold for each set of parallel comparisons (e.g., comparison of the risk of vitamin D deficiency during different gestational age, the adjusted threshold was set at *P* < 0.01). For secondary gestational complications (4 complications) and secondary parturition outcomes (8 outcomes) were exploratory, designed to generate hypotheses rather than test pre-specified research questions. We maintained the significance threshold at α = 0.05 (two-sided) without further adjustment for multiple comparisons.

## Results

### Demographic characteristics and vitamin D status of pregnant women

A total of 2,742 pregnant women with complete records were included. Table 1 presents the demographic characteristics and 25(OH)D concentrations of the participants. The median serum 25(OH)D concentration was 16.3 ng/ml (interquartile range: 10.8–24.9). Of these, 62.5% were vitamin D deficient, 21.2% insufficient, and only 16.3% sufficient. 25(OH)D3 constituted the majority of total vitamin D (median: 14.2 ng/ml), while 25(OH)D2 levels were low (median: 0.6 ng/ml).

**Table 1:** Demographic characteristics and serum 25(OH)D concentrations (ng/ml) of study participants.

**Characteristics**	***n* (%)**	**25(OH)D [M (Q1, Q3)]**
**Sampling season**		
Spring	894 (32.6%)	15.6 (10.2, 24.9)
Summer	744 (27.1%)	16.9 (11.7, 25.4)
Autumn	524 (19.1%)	16.9 (11.9, 25.5)
Winter	580 (21.2%)	15.6 (10.4, 23.7)
**Maternal age (years)**		
20–24	285 (10.4%)	14.2 (9.6, 22.4)
25–29	1,187 (43.3%)	15.5 (10.1, 23.0)
30–34	915 (33.4%)	17.1 (11.7, 27.3)
≥35	355 (12.9%)	18.6 (13.0, 27.1)
**Pre-pregnancy BMI (kg/m** ^2^ **)**		
< 18.5	526 (19.2%)	15.6 (10.2, 23.8)
18.5–23.9	1,943 (70.8%)	16.3 (11.1, 25.1)
≥24.0	273 (10.0%)	16.9 (10.2, 26.5)
**Gestational age (weeks)**		
≤ 12	1,688 (61.6%)	14.0 (9.7, 20.3)
13–24	475 (17.3%)	17.6 (11.1, 28.6)
≥25	579 (21.1%)	25.1 (17.5, 34.8)
**Gravidity**		
1	1,117 (40.7%)	16.0 (10.3, 24.5)
≥2	1,625 (59.3%)	16.4 (11.3, 25.1)
**Parity**		
0	1,698 (61.9%)	16.1 (10.4, 25.3)
≥1	1,044 (38.1%)	16.6 (11.7, 24.3)
**Number of fetuses**		
Singleton	2,671 (97.4%)	16.2 (10.8, 24.6)
Twin	71 (2.6%)	28.1 (16.1, 39.4)
**Pregnancy pattern**		
Natural pregnancy	2,586 (94.3%)	15.8 (10.6, 23.8)
Artificial pregnancy	156 (5.7%)	31.9 (22.4, 39.5)
**Economic status**		
High	429 (15.6%)	16.6 (11.1, 24.4)
Middle	1,684 (61.4%)	16.2 (10.8, 25.0)
Low	629 (23.0%)	15.9 (10.7, 25.2)
**Place of residence**		
City	1,860 (67.8%)	15.9 (10.5, 24.7)
Rural	882 (32.2%)	17.1 (11.7, 25.4)

### Determinants of vitamin D deficiency in pregnant women

A comprehensive subgroup analysis was performed to evaluate potential determinants of vitamin D levels in pregnant women. The variables examined encompassed temporal factors (sampling season), maternal characteristics (age, pre-pregnancy BMI, gestational age, gravidity, parity), pregnancy-related factors (number of fetuses, pregnancy pattern, adverse pregnancy history), environmental influences (economic status, place of residence), nutritional interventions (routine multivitamin supplementation), microbiological factors [group B streptococcus (GBS) colonization], and pre-existing medical conditions (thalassemia, uterine fibroids). Univariate Pearson's chi-square tests were conducted to compare differences in vitamin D status (deficient vs. non-deficient) across subgroups for each variable. The results revealed statistically significant disparities in several variables: maternal age, gestational age, gravidity, number of fetuses, pregnancy pattern, adverse pregnancy history, and routine multivitamin supplementation (all *P* < 0.05). Detailed results of the subgroup analysis are summarized in Table 2.

**Table 2:** Determinants of vitamin D deficiency in pregnant women.

**Variables**	**25(OH)D level**		***χ^2^***	***P***
	<**20 ng/ml [*****n*** **(%)]**	≥**20 ng/ml [*****n*** **(%)]**		
**Sampling season**				
Spring	573 (33.4%)	321 (31.2%)	4.19	0.242
Summer	448 (26.2%)	296 (28.8%)		
Autumn	319 (18.6%)	205 (20.0%)		
Winter	374 (21.8%)	206 (20.0%)		
**Maternal age (years)**				
20–24	202 (11.8%)	83 (8.1%)	30.22	< 0.001
25–29	785 (45.8%)	402 (39.1%)		
30–34	525 (30.6%)	390 (37.9%)		
≥35	202 (11.8%)	153 (14.9%)		
**Pre-pregnancy BMI (kg/m** ^2^ **)**				
< 18.5	344 (20.1%)	182 (17.7%)	2.73	0.256
18.5–23.9	1,206 (70.3%)	737 (71.7%)		
≥24.0	164 (9.6%)	109 (10.6%)		
**Gestational age (weeks)**				
≤ 12	1,257 (73.3%)	431 (41.9%)	332.93	< 0.001
13–24	269 (15.7%)	206 (20.1%)		
≥25	188 (11.0%)	391 (38.0%)		
**Gravidity**				
1	726 (42.4%)	391 (38.0%)	4.97	0.027
≥2	988 (57.6%)	637 (62.0%)		
**Parity**				
0	1,069 (62.4%)	629 (61.2%)	0.38	0.543
≥1	645 (37.6%)	399 (38.8%)		
**Number of fetuses**				
Singleton	1,690 (98.6%)	981 (95.4%)	25.63	< 0.001
Twin	24 (1.4%)	47 (4.6%)		
**Pregnancy pattern**				
Natural pregnancy	1,683 (98.2%)	903 (87.8%)	128.31	< 0.001
Artificial pregnancy	31 (1.8%)	125 (12.2%)		
**Economic status**				
High	268 (15.6%)	161 (15.7%)	2.62	0.270
Middle	1,036 (60.5%)	648 (63.0%)		
Low	410 (23.9%)	219 (21.3%)		
**Place of residence**				
City	1,178 (68.7%)	682 (66.3%)	1.68	0.205
Rural	536 (31.3%)	346 (33.7%)		
**Adverse pregnancy history**				
No	1,459 (85.1%)	799 (77.7%)	24.20	< 0.001
Yes	255 (14.9%)	229 (22.3%)		
**Routine multivitamin supplementation**				
No	1,346 (78.5%)	370 (36.0%)	496.54	< 0.001
Yes	368 (21.5%)	658 (64.0%)		
**Pregnancy with GBS colonization**				
No	1,581 (92.2%)	957 (93.1%)	0.68	0.452
Yes	133 (7.8%)	71 (6.9%)		
**Pre-pregnancy with uterine fibroids**				
No	1,636 (95.4%)	970 (94.4%)	1.63	0.205
Yes	78 (4.6%)	58 (5.6%)		
**Pre-pregnancy with thalassemia**				
No	1,606 (93.7%)	978 (95.1%)	2.44	0.128
Yes	108 (6.3%)	50 (4.9%)		

### Risk factors for vitamin D deficiency in pregnant women

To identify independent risk factors for vitamin D deficiency in pregnant women, a multivariate logistic regression analysis was conducted with vitamin D deficiency (dependent variable: deficient = 1, non-deficient = 0) and the seven variables that showed statistical significance (*P* < 0.05) in the univariate subgroup analysis as independent variables. The model was adjusted for potential confounding factors (maternal age, pre-pregnancy BMI, residential location, sampling season) as predefined in the Methods section, and odds ratios (OR) with corresponding 95% confidence intervals (95% CI) were calculated.

The multivariate logistic regression results indicated that gestational age ≤ 24 weeks, natural pregnancy, absence of prior adverse pregnancy history, and non-adherence to routine multivitamin supplementation were independent risk factors for vitamin D deficiency (all *P* < 0.05). Further subgroup analysis stratified by gestational periods demonstrated that the risk of vitamin D deficiency in early and middle pregnancy was 3.37-fold and 2.08-fold higher. Detailed results of the multivariate logistic regression and stratified analysis are presented in Table 3.

**Table 3:** Analysis of independent risk factors for vitamin D deficiency in pregnant women.

**Variable**	***P***	**OR**	**95% CI**
**Maternal age (years)**			
20–24	0.060	1.47	0.99–2.18
25–29	0.429	1.13	0.84–1.51
30–34	0.815	0.97	0.73–1.29
≥35			Reference
**Gestational age (weeks)**			
≤ 12	< 0.001	3.37	2.67–4.26
13–24	< 0.001	2.08	1.58–2.74
≥25			Reference
**Gravidity**			
1	0.493	1.08	0.88–1.34
≥2			Reference
**Number of fetus**			
Singleton	0.401	1.31	0.70–2.45
Twin			Reference
**Pregnancy pattern**			
Natural pregnancy	< 0.001	5.07	3.20–8.04
Artificial pregnancy			Reference
**Adverse pregnancy history**			
No	0.028	1.32	1.03–1.70
Yes			Reference
**Routine multivitamin supplementation**			
No	< 0.001	4.18	3.46–5.05
Yes			Reference

### Association between early pregnancy vitamin D deficiency and the incidence of common gestational complications

Serum 25(OH)D levels were quantified via LC-MS/MS in 1,688 pregnant women during early pregnancy ( ≤ 12 weeks). Among these participants, 1,257 (74.5%) met the criteria for vitamin D deficiency. During the entire gestational period, the incidence of common complications was as follows: gestational hypothyroidism (*n* = 371, 22.0%), gestational diabetes mellitus (*n* = 326, 19.3%), gestational anemia (*n* = 132, 7.8%), intrahepatic cholestasis of pregnancy (*n* = 105, 6.2%), and gestational hypertension (*n* = 80, 4.7%).

For the pre-specified primary gestational complication outcome (gestational hypothyroidism), early pregnancy vitamin D deficiency was associated with a 32% increased risk of gestational hypothyroidism (OR = 1.32, 95% CI: 1.01–1.74, *P* = 0.048). This finding aligns with the hypothesized vitamin D-thyroid axis interaction and supports the biological plausibility of the association. Exploratory secondary gestational complication outcomes (gestational diabetes mellitus, gestational anemia, intrahepatic cholestasis of pregnancy, gestational hypertension) showed no significant associations with early pregnancy vitamin D deficiency. Detailed results are summarized in Table 4.

**Table 4:** Association between early pregnancy vitamin D deficiency and the incidence of gestational complications.

**Gestational complications**	**Vitamin D deficiency**	**Unadjusted**			**Adjusted**		
		***P***	**OR**	**95% CI**	***P***	**OR**	**95% CI**
**Gestational hypothyroidism [*****n*** **(%)]**							
291 (23.2%)	Yes	0.048	1.32	1.00–1.74	0.048	1.32	1.01–1.74
80 (18.6%)	No	Reference			Reference		
**Gestational diabetes mellitus [*****n*** **(%)]**							
237 (18.9%)	Yes	0.415	0.89	0.68–1.17	0.941	0.99	0.75–1.31
89 (20.7%)	No	Reference			Reference		
**Gestational anemia [*****n*** **(%)]**							
107 (8.5%)	Yes	0.072	1.51	0.96–2.37	0.056	1.55	0.99–2.44
25 (5.8%)	No	Reference			Reference		
**Intrahepatic cholestasis of pregnancy [*****n*** **(%)]**							
82 (6.5%)	Yes	0.379	1.24	0.77–2.00	0.380	1.24	0.77–2.00
23 (5.3%)	No	Reference			Reference		
**Gestational hypertension [*****n*** **(%)]**							
60 (4.8%)	Yes	0.911	1.03	0.61–1.73	0.802	1.07	0.64–1.80
20 (4.6%)	No	Reference			Reference		

### Association between late pregnancy vitamin D deficiency and parturition outcomes

A cohort of 579 pregnant women underwent initial vitamin D assessment during late pregnancy (≥25 weeks), revealing 188 participants (32.5%) of vitamin D deficiency. For the pre-specified parturition outcome (preterm birth), late pregnancy vitamin D deficiency was inversely associated with preterm birth (OR = 0.32, 95% CI: 0.13–0.79, *P* = 0.014). This paradoxical finding conflicts with most biological hypotheses and requires further causal validation. Exploratory secondary outcomes (fetal distress, low birth weight, etc.) showed no significant associations with late pregnancy vitamin D deficiency. Detailed results are presented in Table 5.

**Table 5:** Association between late pregnancy vitamin D deficiency and adverse parturition outcomes.

**Parturition outcomes**	**Vitamin D deficiency**	**Unadjusted**			**Adjusted**		
		***P***	**OR**	**95% CI**	***P***	**OR**	**95% CI**
**Maternal adverse outcomes**							
**Uterine atony [*****n*** **(%)]**							
108 (57.5%)	Yes	0.366	1.17	0.83–1.67	0.111	1.35	0.93–1.94
209 (53.5%)	No	Reference			Reference		
**Cesarean delivery [*****n*** **(%)]**							
103 (54.8%)	Yes	0.555	1.11	0.78–1.58	0.166	1.30	0.90–1.88
204 (52.2%)	No	Reference			Reference		
**Postpartum hemorrhage [*****n*** **(%)]**							
9 (4.8%)	Yes	0.229	1.74	0.71–4.27	0.160	1.92	0.77–4.78
11 (2.8%)	No	Reference			Reference		
**Postpartum anemia [*****n*** **(%)]**							
26 (13.8%)	Yes	0.481	1.20	0.72–2.02	0.443	1.23	0.73–2.07
46 (11.8%)	No	Reference			Reference		
**Oligohydramnios [*****n*** **(%)]**							
17 (9.0%)	Yes	0.649	1.15	0.62–2.14	0.776	1.10	0.59–2.05
31 (7.9%)	No	Reference			Reference		
**Neonatal adverse outcomes**							
**Prematurerupture of membrane [*****n*** **(%)]**							
44 (23.4%)	Yes	0.918	1.02	0.68–1.54	0.866	0.97	0.64–1.47
90 (23.0%)	No	Reference			Reference		
**Preterm birth [*****n*** **(%)]**							
6 (3.2%)	Yes	0.005	0.28	0.18–0.68	0.014	0.32	0.13–0.79
41(10.5%)	No	Reference			Reference		
**Fetal distress [*****n*** **(%)]**							
14 (7.5%)	Yes	0.093	1.89	0.90–3.95	0.139	1.77	0.83–3.79
16 (4.1%)	No	Reference			Reference		
**Term low birth weight [*****n*** **(%)]**							
7 (3.7%)	Yes	0.947	0.97	0.39–2.42	0.850	0.92	0.36–2.31
15 (3.8%)	No	Reference			Reference		

### Comparative analysis of the efficacy of various vitamin D supplementation regimens

A retrospective cohort study was conducted to evaluate the efficacy of vitamin D supplementation in pregnant women diagnosed with vitamin D deficiency during early pregnancy. The study participants adhered to their regular dietary patterns while concurrently following a prescribed vitamin D supplementation regimen. The study cohort comprised 1,303 participants, stratified into five distinct groups based on the formulation and daily dosage of vitamin D supplementation: (1) D2 400 IU/day, (2) D2 800 IU/day, (3) D3 400 IU/day, (4) D3 800 IU/day, and (5) D2 400 IU/day + D3 400 IU/day.

**Figure 1:**
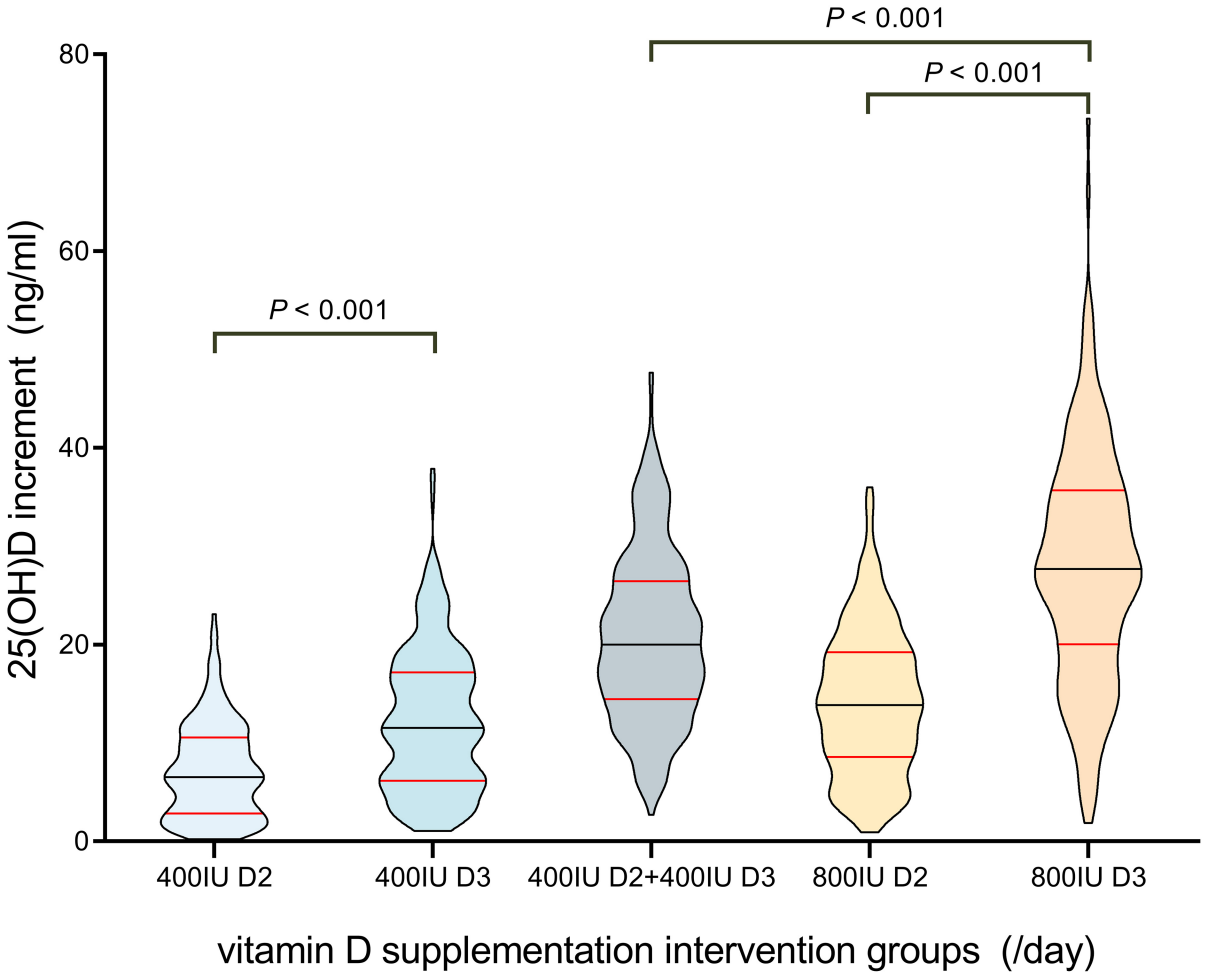
Violin plot of 25(OH)D level increments across vitamin D supplementation intervention groups. This plot depicts the distribution of 25(OH)D increments (unit: ng/ml) in five groups: 400IU D2, 400IU D3, 400IU D2 + 400IU D3, 800IU D2, and 800IU D3. Data are presented as median (central black horizontal line) and interquartile range (red horizontal lines).

Serum 25(OH)D concentrations were quantified via LC-MS/MS at baseline and following a one-month supplementation period. The pre- and post-intervention 25(OH)D levels are presented in Table 6, while the distribution and intergroup comparisons of 25(OH)D level increments (post-intervention minus baseline) are illustrated in Figure 1. Statistical analysis employing the Kruskal–Wallis H test demonstrated that the increment in 25(OH)D levels was significantly greater in the D3 400 IU/day cohort compared to the D2 400 IU/day cohort (*P* < 0.001). Moreover, the D3 800 IU/day regimen elicited a significantly higher 25(OH)D increment than both the D2 800 IU/day regimen and the combined D2 400 IU/day + D3 400 IU/day regimen (*P* < 0.001 for both comparisons).

**Table 6:** Alterations in serum 25(OH)D concentrations (ng/ml) following vitamin D supplementation intervention.

**Group (/day)**	***n***	**Pre-intervention**			**Post-intervention**		
		**25(OH)D2 [M (Q1, Q3)]**	**25(OH)D3 [M (Q1, Q3)]**	**25(OH)D [M (Q1, Q3)]**	**25(OH)D2 [M (Q1, Q3)]**	**25(OH)D3 [M (Q1, Q3)]**	**25(OH)D [M (Q1, Q3)]**
400 IU D2	192	0.5 (0.4, 0.8)	13.6 (9.8, 18.2)	14.6 (10.4, 19.0)	8.5 (5.9, 12.2)	12.4 (8.9, 16.8)	21.6 (16.5, 26.4)
400 IU D3	294	0.5 (0.3, 0.9)	12.6 (8.6, 16.9)	13.5 (9.3, 18.0)	0.7 (0.5, 1.2)	24.2 (19.1, 30.8)	25.5 (20.3, 31.8)
800 IU D2	254	0.5 (0.4, 0.9)	11.2 (8.6, 15.3)	12.4 (9.4, 16.2)	15.0 (11.1, 19.4)	10.9 (8.4, 14.5)	27.0 (21.4, 33.3)
800 IU D3	186	0.5 (0.3, 0.7)	11.2 (8.3, 15.9)	11.7 (8.8, 16.4)	0.7 (0.4, 1.1)	39.2 (30.2, 49.3)	40.1 (31.5, 50.1)
400 IU D2 + 400 IU D3	377	0.5 (0.4, 0.8)	10.0 (7.2, 13.5)	10.9 (7.7, 14.4)	9.5 (5.9, 14.0)	20.2 (15.6, 26.8)	31.6 (24.9, 38.7)

## Discussion

Vitamin D exerts pleiotropic effects via its bioactive metabolite 1,25(OH)_2_D, with 25(OH)D serving as the gold-standard biomarker for nutritional status due to its stability and long half-life (20). Endogenous cutaneous synthesis (via ultraviolet B radiation) and dietary intake (D2 from plants, D3 from animal sources) are the primary sources, with over 95% of circulating 25(OH)D being 25(OH)D3.

This study is the first to use LC-MS/MS to characterize vitamin D status in pregnant women from Deyang, a region with abundant annual solar radiation but high humidity and frequent cloud cover-climatic factors that may attenuate cutaneous vitamin D synthesis. Our findings showed a median 25(OH)D3 concentration of 14.2 ng/ml and 25(OH)D2 of 0.6 ng/ml in the overall cohort, with D2 supplementation significantly elevating 25(OH)D2 levels (occasionally exceeding 25(OH)D3), highlighting the importance of distinguishing between the two metabolites for accurate status assessment. This discrepancy is particularly pronounced in patients supplementing with D2, as their samples exhibit variable concentrations of 25(OH)D2, resulting in suboptimal correlation and substantial systematic bias in analytical outcomes. Conventional immunoassays (ELISA, chemiluminescence) are widely used in clinical settings but suffer from non-equimolar detection of 25(OH)D2/D3, cross-reactivity with 3-epi-25(OH)D3, and interference from elevated vitamin D binding protein (DBP) in pregnancy (21). In contrast, LC-MS/MS enables precise differentiation of vitamin D metabolites, avoiding such biases-critical for populations on D2 supplementation (9). The US CDC has documented inconsistent results between immunoassays and LC-MS/MS, particularly in D2-supplemented individuals, underscoring the necessity of gold-standard quantification for reliable conclusions (22). Our use of LC-MS/MS ensures accurate characterization of vitamin D status in Deyang's pregnant population, addressing a key limitation of prior Chinese studies relying on immunoassays (23).

Vitamin D deficiency is highly prevalent among Chinese pregnant women, with CNNHS data showing a deficiency rate of 87.4% (median 25(OH)D 13.0 ng/ml) during 2015–2017 (23). In our Deyang cohort, the overall deficiency rate was 62.51%, lower than national data but still a public health concern. This regional disparity may stem from Deyang's lower latitude (31°N) compared to northern China, but offset by localtic factors (high humidity, cloud cover) and reduced outdoor activities among pregnant women (a common practice in Chinese culture to avoid “prenatal discomfort”). Notably, early pregnancy deficiency was 74.5% (1,257/1,688), with a significant inverse correlation between gestational age and deficiency risk-consistent with physiological increases in placental mass and widespread prenatal supplementation (24). This aligns with a study in central Iran, where maternal vitamin D deficiency was negatively associated with gestational age, highlighting the universal trend of improving vitamin D status as pregnancy progresses (25).

Contrast to most literature, we observed no seasonal variation in vitamin D status-likely due to Deyang's minimal annual photoperiod fluctuations and reduced outdoor exposure among pregnant women, which mitigates seasonal differences in cutaneous synthesis. Additionally, pre-existing conditions (uterine fibroids, thalassemia) were not associated with deficiency, consistent with previously reports (26). While univariate analysis suggested better vitamin D status in older women [potentially due to increased health awareness (27)], this was not retained as an independent risk factor in multivariate models. A surprising finding was that twin pregnancies were associated with higher 25(OH)D levels than singletons, contrary to the hypothesis of increased nutritional dem. This aligns with Le et al. (21), and may be explained by elevated DBP levels in twin pregnancies, increased placental mass, or enhanced dietary intake. However, the small subgroup size (*n* = 71) limits the generalizability of this result, warranting cautious interpretation.

Our pre-specified primary analysis revealed that early pregnancy vitamin D deficiency was associated with a 32% increased risk of gestational hypothyroidism. This finding is biologically plausible, as vitamin D receptors are expressed in thyroid tissue (14) and vitamin D deficiency has been shown to impair immune tolerance, potentially increasing the risk of autoimmune thyroid disorders (28). The near-significant association supports the need for larger prospective studies to confirm this relationship, particularly given the high co-prevalence of both conditions in Chinese pregnant women and the potential clinical implications for targeted supplementation. For the pre-specified primary outcome of preterm birth, we observed a counterintuitive inverse association with late pregnancy vitamin D deficiency. This finding conflicts with most biological hypotheses—vitamin D is known to regulate inflammatory pathways and uterine smooth muscle function, which are critical for maintaining gestation (17). Potential explanations for this inverse association include: (a) fetal skeletal development delay in deficient mothers—given vitamin D is the sole source for fetal calcium absorption and skeletal mineralization, late pregnancy deficiency may impede fetal skeletal maturation, necessitating longer gestation for complete development; (b) residual confounding from unmeasured factors such as maternal inflammatory cytokines (e.g., TNF-α, IL-6), whose interaction with vitamin D status could modulate preterm risk; and (c) reverse causation, wherein women identified as at high risk of preterm birth may be more likely to receive vitamin D supplementation, thereby increasing their 25(OH)D levels and masking a potential positive association. Sensitivity analysis excluding twins did not alter the core results, but key limitations persist: selection bias cannot be ruled out, as women with severe vitamin D deficiency may have been lost to follow-up or had unrecorded outcomes; critical details were not collected, including fetal skeletal maturation markers (e.g., ultrasound bone measurements) that could validate the proposed developmental delay mechanism; and statistically, we did not adjust for potential confounders such as dietary calcium intake, sunlight exposure, or prenatal supplement use (beyond what was captured in baseline data), nor did we perform subgroup analyses by the severity of vitamin D deficiency or maternal comorbidities-gaps that could clarify the specificity and generalizability of the observed inverse association.

Historically, vitamin D2 and D3 were deemed equipotent, leading to public health recommendations that did not distinguish between the two forms (29, 30). However, emerging research has highlighted disparities in their potency and metabolic efficacy: D3 exhibits superior VDR binding affinity and greater efficacy in maintaining circulating 25(OH)D concentrations (31, 32). A seminal study by Armas et al. (33), elucidated serum 25(OH)D kinetics following single-dose administration, showing D3 was substantially more potent than D2 in elevating and sustaining 25(OH)D levels—with a differential efficacy of at least threefold. Notably, the comparative efficacy of D2 and D3 in maintaining 25(OH)D during pregnancy remains incompletely understood. Our investigation demonstrated that D3—administered at an equivalent dosage to D2 or in combination with it—was superior to D2 monotherapy in augmenting 25(OH)D levels. While D2 monotherapy significantly increased serum 25(OH)D2, this was accompanied by a reciprocal decrease in 25(OH)D3 concentrations, partially explaining D2's reduced efficacy relative to D3. We therefore advocate for D3 as the optimal form of vitamin D supplementation during gestation. Additionally, our findings revealed an inverse correlation between baseline 25(OH)D concentrations at initial assessment and the magnitude of 25(OH)D elevation following supplementation.

## Conclusion

Vitamin D deficiency is highly prevalent (62.5%) among pregnant women in Deyang, southwest China, with early pregnancy ( ≤ 12 weeks) representing the highest-risk window. Based on pre-specified primary outcomes, early pregnancy vitamin D deficiency was associated with an increased risk of gestational hypothyroidism, while late pregnancy vitamin D deficiency showed a significant inverse association with preterm birth. These findings highlight the complex role of vitamin D in pregnancy and the need for targeted research to clarify causal relationships. Given the high deficiency prevalence and its link to gestational hypothyroidism, systematic vitamin D screening is recommended for Deyang's pregnant population in early pregnancy. Targeted supplementation should be prioritized for high-risk groups, including those in early gestation, with natural conception, and non-adherent to multivitamins. Vitamin D3 (400–800 IU/day) is the preferred form for correcting deficiency. Integrating vitamin D screening into routine prenatal care aligns with global efforts to improve maternal-fetal health. These findings collectively support targeted screening and supplementation strategies to optimize pregnancy outcomes in this region.

## Data Availability

The original contributions presented in the study are included in the article/supplementary material, further inquiries can be directed to the corresponding author/s.
